# Temporomandibular disorder, body pain and systemic diseases: assessing their associations in adolescents

**DOI:** 10.1590/1678-7757-2019-0608

**Published:** 2020-09-07

**Authors:** Guilherme Vinícius do Vale BRAIDO, Leticia Bueno CAMPI, Paula Cristina JORDANI, Giovana FERNANDES, Daniela Aparecida de Godoi GONÇALVES

**Affiliations:** 1 Universidade Estadual Paulista Faculdade de Odontologia Departamento de Materiais Dentários e Prótese AraraquaraSão Paulo Brasil Universidade Estadual Paulista (UNESP), Faculdade de Odontologia, Departamento de Materiais Dentários e Prótese, Araraquara, São Paulo, Brasil.

**Keywords:** Adolescent, Facial pain, Temporomandibular joint dysfunction syndrome

## Abstract

Painful temporomandibular disorders (TMD) in children and adolescents may impact negatively the individual´s life. The presence of comorbidities associated with TMD tends to increase the persistence of pain and to facilitate its chronification.

**Objective:**

To investigate the presence of other painful conditions and systemic diseases and their association with painful TMD.

**Methodology:**

In this cross-sectional study, 690 adolescents aged between 12-14 years old were evaluated through questionnaires and clinical examinations.

**Results:**

Painful TMD was found in 16.2% of the sample, with a significant association with bronchitis (OR= 2.5; p=0.003) and asthma (OR=3.1; p=0.013), reported by the parents/legal guardians of the participants. Adolescents with regional and widespread pain were 2.7 (95% CI: 1.65-4.55) and 3.6 (95% CI: 1.29-10.14) more likely to also present painful TMD. Painful TMD was associated with a higher number of body pain sites in the last 12 months (4.26 vs. 2.90; p<0.001), as well as a higher number of systemic diseases (1.48 vs. 1.18; p=0.048), when compared to adolescents without painful TMD.

**Conclusion:**

The findings of this study point out the importance of considering the presence of comorbid conditions in the diagnosis and management of painful TMD in adolescents. A multidisciplinary approach would contribute to better control of painful TMD and decrease its chronification risk.

## Introduction

Adolescence is a complex phase of life when individuals experience intense emotional, cognitive, social, physical, and hormonal transformations. It is also the period in which the behavioral traits are defined.^1^ Occurrence of pain at this stage can be associated with disability and a predictive factor for pain in adulthood.^2^ Anxiety, depression, reduced quality of life, and school absenteeism are conditions frequently present in adolescents with somatic pain.^3^ These experiences represent significant risk factors for the development of chronic pain in the later stages of life. Therefore, a better understanding of pain features in this age group is fundamental to define more efficient treatment protocols as well as to prevent future health problems.^2,4^

Temporomandibular disorders (TMD) are defined as a group of musculoskeletal and neuromuscular conditions involving temporomandibular joints (TMJs), masticatory muscles, and associated structures. Pain is the most frequent symptom and can affect both muscles or joints.^5^ Although TMD has been more investigated in adults than in adolescents, recent studies have pointed to an increase in its prevalence among the latter, with rates ranging from 7.3% and 30.4%.^6^

The complexity of TMD is enhanced by its association with other painful conditions as headaches, neck pain, and back pain.^7^ Moreover, the emotional and psychosocial aspects can influence the painful experience related to TMD.^8,9^ Previous studies investigated the prevalence of headache, abdominal pain, low back pain and TMD in children and adolescents,^10-12^ and have reported prevalence rates similar to those observed among adults. The association between painful TMD and some systemic conditions, such as cardiac diseases^13,14^ and rheumatic diseases,^15^ have been demonstrated in adult populations. Since there is a lack of similar studies in adolescents, it is of high interest to investigate the interaction among these conditions. The objective of this study was to investigate the relationship between painful TMD and comorbid conditions in adolescents, including pain in other areas and systemic diseases, after identification and adjustment of confounding factors. Increasing the knowledge about the prevalence of painful TMD, as well as the existence of comorbid conditions in adolescents, is essential for prevention and to develop treatment strategies, aiming to reduce the pain and suffering, as well as the risk of developing chronic pain in the adult life.^4^

## Methodology

The sample of this cross-sectional study was composed of adolescents aged between 12-14 years old, enrolled in public and private schools. Participants were informed about the objectives of the study and were invited to participate in the evaluation. We only included in the sample, those who presented the free and informed consent form signed by their parents or legal guardian, and a consent form signed by themselves.

This study was approved by the Research Ethics Committee of São Paulo State University (Unesp), School of Dentistry, Araraquara (CAAE number: 54755616.3.0000.5416). Authorizations were obtained from the Education Department of the City of Araraquara and the boards of each school participating in this study.

### Pilot Study e Sample Size

Before starting the data collection, a previous pilot study was conducted in a sample of 149 adolescents (13-15 years old). The Research Diagnostic Criteria for Temporomandibular Disorders (RDC/TMD) was used to classify the TMD cases, and the Standardized Nordic Questionnaire was applied to assess the presence of body pain. We found that 72.7% of the volunteers free of TMD reported persistent pain in at least one area of the body (excluding the face, head, neck, and shoulders), while among individuals presenting painful TMD, the prevalence was 90.4%.

The sample size was determined using the software from the Statistics Department of the University of British Columbia, Canada (http://www.stat.ubc.ca/~rollin/stats/ssize/b2.html). The present study was part of a broader project that aimed to evaluate the association of TMD (outcome variable) with other conditions (predictor variables), including fibromyalgia, obesity, persistent body pain, primary headaches, sleep problems, and symptoms of depression and anxiety. The main project is entitled “Epidemiological investigation of the relationship between temporomandibular disorders, painful comorbidities, obesity and sleep disorders in adolescents”. So far, two previous papers were published, presenting part of the collected data.^16,17^ Each of them, although based on the same sample, had specific aims, and consequently, different instruments and evaluations were applied. For this reason, it is recommended to calculate the sample size separately for each of the primary outcomes by applying a Bonferroni correction to adjust the significance level. Thus, for the sample size calculation, we considered the prevalence obtained in the pilot study, a statistical power to refute H0 of 80%, and alpha of 0.007, adopted after Bonferroni correction, according to our goals.^18^ Accordingly, the sample should consist of approximately 120 individuals with painful TMD and 120 individuals free of painful TMD. Also, we added 25% due to possible losses during the data collection, thus totaling a minimum of 300 participants in the study. The investigation of the association between systemic diseases and painful TMD was an exploratory analysis.

### Assessment instruments

The family economic classification was determined according to the updated Brazilian Classification Criteria,^19^ through a standardized questionnaire considering the household characteristics and the educational level of the head of the family. For the analyses, the strata were grouped into three classifications: 1) High (A1, A2, B1, and B2); 2) Medium (C1 and C2) and 3) Low (D / E).

A standardized questionnaire assessing the orofacial pain characteristics (location, intensity, quality, duration, and factors causing aggravation or improvement of the pain) was applied for initial diagnosis of the TMD and the American Academy of Orofacial Pain Diagnostic Criteria^5^ was applied to the differential diagnoses of other conditions that may mimic painful TMD. We also conducted an intraoral examination to exclude the presence of intraoral painful conditions such as caries, mucosal lesions, and periodontal disease.

To classify the TMD, we applied the Brazilian version of the Research Diagnostic Criteria for Temporomandibular Disorders (RDC/TMD), axis I.^20^ The following questions from RDC/TMD Axis II questionnaire, were added: #3 (“Have you had pain in the face, jaw, temple, in front of the ear or in the ear in the past month?”), #4a (“How many years ago did your facial pain begin?”) or #4b (“How many months ago did your facial pain begin?”), 14a# (“Have you ever had your jaw lock so that it wouldn’t open all the way?”), and, if yes, 14b# (“Was this limitation in jaw opening severe enough to interfere with your ability to eat?). The painful TMD group was composed by adolescents presenting TMD classified as Group I (myofascial pain with or without limited mouth opening) and/or Group III - Axis I (TMJ arthralgia, and/or TMD osteoarthritis). The researcher who conducted the physical examination was blinded for any other variables collected.

The assessment of body pain was done through the Standardized Nordic Questionnaire, translated and validated to Brazilian Portuguese.^21^ It is an instrument that allows a standardized assessment of musculoskeletal pain, and an effective tool of easy interpretation indicated for individuals aged between 10 to 19 years-old.^22^ Additionally, individuals are questioned about the occurrence of pain and/or tingling/numbness in each of the 9 different areas of the body in the last 12 months and in the last 7 days. The body areas were grouped in regional pain (neck/shoulder) and widespread pain (upper back, elbows, wrists/hands, lower back, hips/thighs, knees and ankles/feet).^23^

Parents or legal guardians of adolescents responded a questionnaire about the current medical condition, considering a previous medical diagnosis. This information was collected and evaluated in a list, which was presented to legal guardians, containing the most frequent diseases reported among Brazilian youth.^24^ These diseases were grouped as follows: 1) Respiratory diseases (bronchitis, rhinitis, asthma, sinusitis, pulmonary emphysema, and pneumonia); 2) Gastrointestinal diseases (gastro-esophageal reflux; irritable bowel syndrome, constipation, gastritis/ulcer, and colitis); 3) Endocrine diseases (diabetes, hypothyroidism, hyperthyroidism, and hypoglycemia); 4) Diseases which may include painful conditions (fibromyalgia, rheumatoid arthritis, erythematous lupus, and migraines); 5) Cardiac/Hematologic Diseases (anemia, hypertension, cholesterol, and heart problems); 6) Psychological conditions (depression, anorexia/bulimia, and attention deficit hyperactivity disorder); and 7) Gynecological diseases (polycystic ovary and endometriosis).

The evaluation of sexual maturation was based on the five stages of pubic hair development, proposed by Tanner in 1962, and later adapted for self-evaluation through the use of drawings.^25^ Self-evaluation of pubic hairiness showed a satisfactory agreement with medical assessment and is adequate to determine the stage of sexual maturation in both genders.^25^ Participants were assessed individually in a private place, and drawings were shown on a board by a researcher of the same gender. They were instructed to identify the most similar picture to their current stage of sexual maturation. The five stages of maturation were grouped in three pubertal development stage (prepubertal, pubertal and postpubertal).

### Exclusion criteria

The exclusion criteria included 1) the presence of tooth pain (e.g., pulpitis, extensive caries) or acute facial pain after recent injury (e.g., macro trauma); 2) altered cognitive functions, impairment of the communication abilities, or difficulties to understand the questionnaires; 3) use of pain medication or substances that can interfere with the central nervous system; 4) current orthodontic or TMD treatment.

### Statistical methods and data analysis

For data analysis purposes, we considered the painful TMD as the main outcome variable. Systemic diseases, and body pain sites as the independent variables, and sociodemographic characteristics (age, gender, pubertal developmental stage, and income class) were considered possible confounder variables. Descriptive statistics and frequency counts were used to characterize the sample. A single logistic regression was conducted to study the association between painful TMD and systemic diseases and painful TMD and body pain sites. Afterwards, multiple logistic regression models analyses, adjusted by confounder variables, were performed to study the association between painful TMD and body pain sites and systemic diseases. Also, a single linear regression model analyses were performed for the association between painful TMD and the number of body pain sites and the number of systemic diseases. Then, multiple linear regression models analyses, adjusted by confounder variables, were performed to study the association between painful TMD and body pain sites and systemic diseases. The adopted significance level was 0.05. All analyses were performed with the SPSS software package, version 21.0, for Mac.

## Results

**Figure1:**
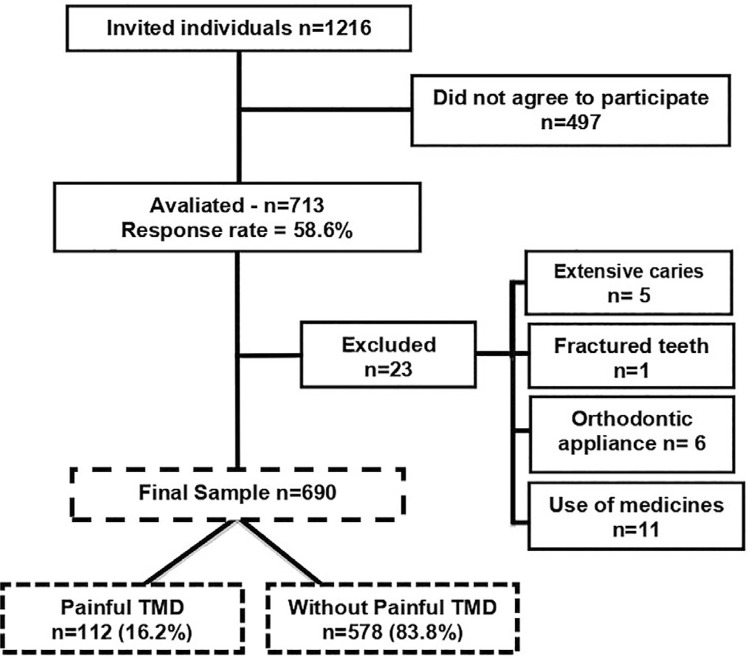
Sample flowchart

A total of 1,216 adolescents were invited to participate in the study. Of them, 713 agreed to participate and returned the written term of consent (a response rate of 58.6%). According to the exclusion criteria, 23 adolescents were excluded (Figure 1): 5 due to extensive caries, 1 with a fractured tooth, 6 due to orthodontic appliance use, and 11 due to medication use (Methylphenidate and Lisdexamfetamine, Flunarizine dihydrochloride, Phenobarbital, Clomipramine hydrochloride, Prednisone, Lorazepam, and Topiramate). The present study was composed of 690 adolescents. A *post-hoc* test showed that our sample had a power of 0.9 (α=0.05; effect size=0.3) for the association between body pain sites and painful TMD. Although the study regarding the association between painful TMD and systemic diseases was exploratory, our sample had an adequate sample size with a large effect size (p=0.05). and a relevant power (p=0.08).

Our final sample consisted of 389 girls (56.4%), with most of the adolescents in the pubertal development stage (64.2%), mean age of 12.7±0.74 years and classified as medium income class (48.7%). TMD pain was identified in 112 (16.2%) adolescents. (Table 1)

**Table 1:** Descriptive statistics of the predictor variables stratified by the presence of painful TMD

		Painful TMD^**¶**^		
		No	Yes	Total
Age	Mean	12.70	12.77	12.71
	SD^§^±	0.75	0.78	0.76
	Min. - Max	12.0 - 14.0	12.0 - 14.0	12.0 - 14.0
		**n(%)**	**n(%)**	**n(%)**
Gender	Boy	261 (45.2)	40 (35.7)	301 (46.6)
	Girl	317 (54.8)	72 (64.3)	389 (56.4)
Pubertal development stage	Prepubertal	126(21.8)	26(23.2)	152(22.0)
	Pubertal	376(65.1)	67(59.8)	443(64.2)
	Postpubertal	76(13.1)	19(17.0)	95(13.8)
	High (A/B)	248(42.9)	53 (47.3)	301(43.6)
Income class	Middle (C)	285 (49.3)	51 (45.5)	336(48.7)
	Low (D/E)	44 (7.6)	8 (7.1)	52(7.5)
	Missing	1(0.2)	0(0)	1(0.1)

^¶^TMD=Temporomandibular disorder; ^§^SD=Standard deviation ; *Chi-square test

Among the systemic diseases self-reported by parents/legal guardian, 313 adolescents (45.5%) had respiratory diseases, 78 (11.3%) gastrointestinal diseases, 16 (2.3%) endocrine diseases, 56 (8.1%) heart/hematological diseases, 100 (14.5%) presented conditions related to pain in other areas, 51 (7.4%) emotional/psychological changes and 5 (0.7%) gynecological diseases. In the multiple logistic regression model, only bronchitis and asthma (p<0.05) were statistically associated with painful TMD. In the presence of bronchitis and asthma, individuals presented an increased risk of 2.5 and 3.1 of having painful TMD compared with individuals free of these respiratory conditions (Table 2).

**Table 2:** Logistic regression model for the association between painful TMD and systemic diseases

		Painful TMD^**¶**^			Single Logistic Regression			Multiple Logistic Regression*		
		No n(%)	Yes n(%)	Total n(%)	*p* value	OR^**§**^	95% CI^**†**^	*p* value	OR^**§**^	95% CI^**†**^
**Respiratory diseases**										
Bronchitis	No	531(92.0)	94(83.9)	625(90.6)	Reference			Reference		
	Yes	46(8.0)	18(16.1)	64(9.3)	0.007	2.2	1.23-3.98	0.003	2.5	1.34-4.48
	Missing	1(0.1)	0(0)	1(0.1)						
Asthma	No	562(97.4)	104(92.9)	666(96.5)	Reference			Reference		
	Yes	15(2.6)	8(7.1)	23(3.3)	0.014	2.8	1.19-6.97	0.013	3.1	1.27-7.60
	Missing	1(0.2)	0(0)	1(0.1)						

^¶^TMD=Temporomandibular disorders; ^§^OR=Odds ratio; ^†^CI=confidential interval; *Adjusted model by age, gender, pubertal develoment stage, and income class.

Adolescents reporting regional pain were 2.7 (95% CI: 1.65-4.55) more likely to present painful TMD when compared to those who did not report regional pain. When the areas of regional pain were individually analyzed, pain in the neck (OR=2.4; 95% CI: 1.57-3.70) and in the shoulders (OR=1.7; 95% CI: 1.12-2.57) were statistically associated with painful TMD. Moreover, in the presence of widespread body pain, the risk of presenting painful TMD was 3.6-fold increased, showing a significant association between both conditions. The magnitude of association between painful TMD and specific body areas varied, with upper back presenting the higher magnitude (OR=2.8, 95% CI: 1.84-4.30), followed by wrists/hands (OR=2.4; 1.58-3.62), hip/thighs (OR=2.1; 1.39-3.31), knees (OR=2.0; 1.29-2.98), ankle/feet (OR= 1.8; 1.21-2.78). (Table 3). Moreover, adolescents presenting painful TMD reported a significantly higher number of body pain areas in the last 12 months compared with adolescents with no painful TMD (p<0.001). They also reported a significantly higher number of systemic diseases (p=0.048) (Table 4).

**Table 3:** Logistic regression model for the association between painful TMD and body pain sites

		Painful TMD^**¶**^			Single Logistic Regression			Multiple Logistic Regression*		
		No n(%)	Yes n(%)	Total n(%)	*p* value	OR^**§**^	95% CI^**†**^	*p* value^**£**^	OR^**§**^	95% CI^**†**^
Regional Pain in the last 12 months	No	225(38.9)	21(18.8)	246(35.7)	Reference			Reference		
	Yes	353(61.1)	91(81.3)	444(64.3)	<0.001*	2.8	1.67-4.56	**<0.001**	2.7	1.65-4.55
Neck	No	320(55.4)	38(33.9)	358(51.9)	Reference			Reference		
	Yes	258(44.6)	74(66.1)	332(48.1)	<0.001*	2.4	1.58-3.69	**<0.001**	2.4	1.57-3.70
Shoulders	No	385(66.6)	60(53.6)	445(64.5)	Reference			Reference		
	Yes	193(33.4)	52(46.4)	245(35.5)	0.008*	1.7	1.14-2.60	**0.012**	1.7	1.12-2.57
Widespread Pain in the last 12 months	No	70(12.1)	4(3.6)	74(10.7)	Reference			Reference		
	Yes	508(87.9)	108(96.4)	616(89.3)	**0.007#**	3.7	1.33-10.4	**<0.015**	3.6	1.29-10.1
Upper back	No	358(61.9)	40(35.7)	398(57.7)	Reference			Reference		
	Yes	220(38.1)	72(64.3)	292(42.3)	<0.001*	2.9	1.92-4.46	**<0.001**	2.8	1.84-4.30
Elbow	No	533(92.9)	105(93.8)	638(92.5)	Reference			Reference		
	Yes	45(7.8)	7(6.3)	52(7.5)	0.573*	0.8	0.35-1.80	0.717	0.9	0.37-1.97
Wrists/hands	No	382(66.1)	50(44.6)	432(62.6)	Reference			Reference		
	Yes	196(33.9)	62(55.4)	258(37.4)	<0.001*	2.4	1.60-3.64	**<0.001**	2.4	1.58-3.62
Low back	No	368(63.7)	59(52.7)	427(61.9)	Reference			Reference		
	Yes	210(36.3)	53(47.3)	263(38.1)	0.028*	1.6	1.04-2.37	0.065	1.9	0.98-2.25
Hip/Thighs	No	452(78.2)	70(62.5)	522(75.7)	Reference			Reference		
	Yes	126(21.8)	42(37.5)	168(24.3)	<0.001*	2.1	1.40-3.31	**0.001**	2.1	1.39-3.31
Knees	No	367(63.5)	55(49.1)	422(61.2)	Reference			Reference		
	Yes	211(36.5)	57(50.9)	268(38.8)	0.004*	1.8	1.20-2.71	**0.002**	2.0	1.29-2.98
Ankle/feet	No	358(61.9)	54(48.2)	412(59.7)	Reference			Reference		
	Yes	220(38.1)	58(51.8)	278(40.3)	0.007*	1.7	1.16-2.63	**0.004**	1.8	1.21-2.78

^¶^TMD=Temporomandibular disorders; ^§^OR=Odds ratio; ^†^CI=confidential interval; *Adjusted model by age, gender, pubertal development stage, and income class.

**Table 4:** Multiple Linear regression model for the association between painful TMD, number of body pain sites and number of systemic diseases

	Number of body pain sites in the last 12 months			
	b	SE^**§**^	b	*p* value
Constant	1.63	1.16		
Painful TMD^¶^	1.35	0.18	0.27	**<0.001**
Age	0.12	0.19	0.05	0.195
Gender	-0.74	0.14	-0.02	0.591
Pubertal development stage	-0.04	0.12	-0.01	0.711
Income class	0.01	0.02	-0.01	0.979
	**Number of systemic diseases**			
	**b**	**SE**^**§**^	**b**	***p* value**
Constant	1.05	0.98		
Painful TMD^¶^	0.30	0.15	0.08	**0.048**
Age	0.20	0.08	0.01	0.839
Gender	-0.14	0.12	-0.05	0.236
Pubertal development stage	0.10	0.09	0.04	0.325
Income class	-0.02	0.02	-0.05	0.229

^¶^TMD=Temporomandibular disorder; ^§^SE=Standard Error

## Discussion

TMD has been associated with several painful and non-painful conditions in adults.^14,15,23^ Additionally, it has been demonstrated that the presence of comorbidity tends to increase the severity and the disability related to the involved conditions, as well as make worse their prognostic.^7^ Because there is a lack of information regarding the associations between TMD and other conditions among adolescents, our study is justified. Our main findings are: (1) painful TMD, systemic diseases and persistent body pain are highly prevalent among adolescents; (2) among the reported systemic diseases, bronchitis and asthma are significantly associated with painful TMD; (3) the presence of persistent regional and widespread body pain are more prevalent among adolescents presenting painful TMD when comparing with adolescents free of TMD.

The prevalence rate of painful TMD (16.2%) found in the present sample is in accordance with previous studies that have shown rates varying from 7.3% to 30.4% in adolescents^6^. The instrument used for the TMD assessment and the age of the sample can contribute to the variation observed across previous studies. We did not find significant differences regarding the presence of TMD pain between genders, or the pubertal developmental stages.

Previous studies have shown that painful TMD is associated with the presence of pain in other parts of the body.^26,27^ Our findings agree with the literature confirming the association between painful TMD and regional and widespread pain. The association between painful TMD and the persistent regional pain can be related to the neural inputs from the superior cervical region. The stimuli from the afferent neurons converge to the subnucleus caudalis and may result in sensitization of the orofacial structures. Another hypothesis is that peripheral and central sensitization mechanisms may occur which could explain this association, as well as the presence of persistent pain all over the body.^28^

The presence of widespread pain can indicate changes related to central sensitization (CS), explaining the comorbid relationship between painful conditions.^29^ Previous studies pointed out the involvement of CS with chronic painful conditions.^10,30^ CS is characterized by neuroplastic changes in the neurons of the Central Nervous System (CNS), resulting in a reduction of the pain threshold, facilitation for the nociceptive stimuli, and amplification of the receptive fields of these neurons, causing a decrease in neuronal excitability thresholds.^29,31^ This enhanced sensitivity has been attributed to pathological changes in central pain processing such as sensitization of spinal nociceptive neurons and disturbances of descending noxious control systems.^29^ In this state of generalized sensitization, the entrance of noxious stimuli from different sites is poorly modulated, resulting in amplification of pain and increasing the risk of chronification.^28^ TMD is a painful condition and the CS process may play a relevant role in its pathophysiology.^30^ Therefore, it is plausible to hypothesize that CS may explain, at least in part, the association between painful TMD and widespread body pain observed in this sample.

There is a lack in the literature regarding the association between systemic diseases and TMD in adolescents. In adults, previous studies showed an association between the presence of TMD and some specific systemic diseases, such as irritable bowel syndrome,^32^ heart diseases,^13^ and autoimmune diseases, such as rheumatic disease.^15^ In our sample, asthma and bronchitis were significantly associated with painful TMD. We highlight that although we found a significant association between respiratory conditions and the presence of painful TMD, the number of participants affected by both was small. We found 18 adolescents with painful TMD reporting previous diagnosis (informed by their parents or legal guardians) of bronchitis, and eight reporting asthma. Although our results should be interpreted with caution and confirmed in future studies, they are aligned with a previously conducted study, demonstrating a positive correlation between TMD and respiratory diseases in children.^33^ The presence of some degree of inflammation in the respiratory system and in the organism overall seems to be associated with bronchitis and asthma.^34^ One exploratory hypothesis may consider the effect of cytokines in the nervous system structures and functions. It has been shown that cytokines are originated in the immunological, neuronal, and glial cells. The cytokines may cause different effects on the nervous system, such as hyperexcitability and changes in the expression of nociceptors. Overall, the process can result in an exacerbation of painful processes.^35^ These neuronal changes can increase the excitability of the nociceptive neuronal circuits, generating a “functional plasticity” and central sensitization.^28,29^ Moreover, pro and anti-inflammatory cytokines have been associated with the presence of TMD pain and widespread palpation tenderness in the body,^36^ highlighting the influence of these endogenous biomarkers in the physiopathology of painful conditions. Additionally, during asthma attacks, there is excessive use of the accessory respiratory muscles, which can increase the tension in the muscles of the cervical region and more anterior positioning of the head. These changes can generate a nociceptive stimulus on the trigeminocervical system, contributing to orofacial pain.^28,33^

Our study has some limitations. Firstly, since it was a cross-sectional study, causation cannot be inferred. We assessed the presence of systemic diseases through the report of the adolescent’s parents or legal guardian, so no definitive diagnosis was provided, neither the severity of the diseases. Therefore, the gold standard diagnostic tool, which would be an accurate medical examination and assessment conducted within the last 12 months,^37^ was not possible. However, the sample had an adequate size, as described in the results.

Another limitation was regarding the type of school. Most of the sample were enrolled in public schools, due to a low participation response of students from private schools, who did not return the consent form signed by their parents/legal guardians. Finally, it is important to point out that the presence of pain in adolescents is often associated with anxiety and depression symptoms.^8,9^ Given this, future studies should address the interaction of these psychological factors with painful TMD, and the associated variables found in this study.

The study’s strengths are related to instruments and sampling. Translated and validated instruments with easy interpretation for adolescents were applied to evaluate the conditions studied. For TMD classification we applied the RDC/TMD, and the researcher who performed the physical and intraoral exams was blinded for the other variables.

The investigation of the presence of painful TMD and comorbidities in adolescents is highly relevant to improve the current knowledge about the epidemiology and risk factors related to these conditions, and to improve the understanding of the risks associated with chronic pain in adults as well. Moreover, to increase our comprehension can be beneficial to facilitate the development of treatment strategies, including all those conditions, aiming better prognoses and avoiding unnecessary treatments. The presence of pain in adolescents can have a significant impact on their quality of life.

The findings of this study point out the importance of considering the presence of comorbid conditions in the diagnosis and management of painful TMD in adolescents. A multidisciplinary approach would contribute to better control of painful TMD and decrease its chronification risk. Therefore, future studies should be performed to improve our understanding between the mechanisms directly related to the relationship of these conditions and to clarify the possible causal relationship.
